# Supplementary material for: NusG inhibits RNA polymerase backtracking by stabilizing the minimal transcription bubble
Source: eLife. 2016 Oct 4;5:e18096. doi: 10.7554/eLife.18096 (PMC5100998; doi:10.7554/eLife.18096)
Supplement: Supplementary file 1. — DOI: http://dx.doi.org/10.7554/eLife.18096.027 [file elife-18096-supp1.pdf]

Supplementary file 1.
Model employed for fitting of nucleotide addition and translocation data in Figure 2B.

Initial conditions (See Note 1):

T=0   A = p (~0.2)   B = 1-p (~0.8)   C = 0   D = 0

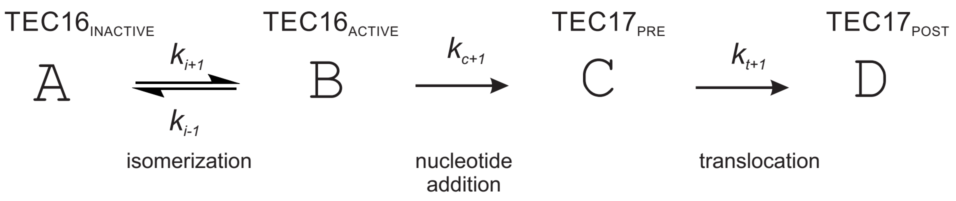

Rate equations (See Note2):

$dA/dt = -A \times k_{i+1} + B \times k_{i-1}$

$dB/dt = -B \times k_{c+1} - B \times k_{i-1} + A \times k_{i+1}$

$dC/dt = -C \times k_{t+1} + B \times k_{c+1}$

$dD/dt = C \times k_{t+1}$

Equations for dependent variables:

RNA17 = F1×(C+D)

SF_fluorescence=F2×(A + B + C) + F3×D

Independent variables: T -time

Dependent variables:
RNA17 -RNA17 band intensities from quench flow experiment
SF_fluorescence -Fluorescent trace from stopped-flow experiment

Parameters:
k_{i+1} -rate of isomerization of inactive TEC into active TEC
k_{i-1} -rate of isomerization of active TEC into inactive TEC
k_{c+1} -rate of nucleotide incorporation
k_{t+1} -rate of forward translocation

F1 -normalization coefficient for quench flow data
F2 -normalization coefficient for stopped-flow data
F3 -normalization coefficient for stopped-flow data
p -fraction of inactive TEC (See Note 1)

Note 1: The fraction of inactive TEC ("p" parameter) is determined by k_{i+1} and k_{i-1} because TEC16 is assembled and reaches equilibrium in the absence of NTP. To reflect this condition we introduced a 100 s mixing step without NTP into the Kintek Explorer virtual experiment setup. The initial fractions of active and inactive TEC16 can then be chosen arbitrary, the "p" parameter is redundant and is not explicitly used.

Note 2: Rate equations are uniquely defined by the reaction scheme and do not need to be explicitly specified when fitting data with Kintek Explorer.
