# Supplementary material for: NusG inhibits RNA polymerase backtracking by stabilizing the minimal transcription bubble
Source: eLife. 2016 Oct 4;5:e18096. doi: 10.7554/eLife.18096 (PMC5100998; doi:10.7554/eLife.18096)
Supplement: Supplementary file 2. — DOI: http://dx.doi.org/10.7554/eLife.18096.028 [file elife-18096-supp2.pdf]

Supplementary file 2.
Model employed for fitting pyrophosphorolysis, backtracking, RNA cleavage and dinucleotide release data in Figures 2C and 3.

Pyrophosphorolysis, backtracking, RNA cleavage, dinucleotide release and data were poorly described by the single exponential function and were fit to the stretched exponential function (also known as the two parameter Weibull cumulative density function). The stretched exponential was first described by Rudolf Kohlrausch (1809 - 1858) in 1854 and is commonly employed for the phenomenological description of heterogeneous and disordered systems (see e.g. Sluch MI, Somoza MM & Berg MA (2002) *J. Phys. Chem.* B 106: 7385–7397). Here the stretched exponential allowed for an adequate empirical fit to data with just two parameters and the robust estimation of the median reaction times.

For normalized data the stretched exponential function is expressed as:

$F(t)= 1-e^{-(k_1 \cdot t)^{k_2}}$ for increasing signal

$F(t)= e^{-(k_1 \cdot t)^{k_2}}$ for decreasing signal

k_2 is the stretching parameter (0.5-0.8 in our systems). Stretching may occur due to the heterogeneity in the TEC population and/or the change in the TEC reactivity over time (aging, e.g. due to backtracking by many nucleotides). The stretched exponential does not discriminate between the above possibilities and instead combines their effect into a single parameter k_2. k_1 corresponds to the initial reactivity (the initial reaction rate constant) if all stretching is due to the changes in the TEC reactivity over time. However, we opted to use the median of the stretched exponential to quantitatively compare the progress of the reactions over time because the median includes both k_1 and k_2 parameters and does not require additional assumptions about the mechanism of the process.

The median reaction times were calculated as:

$$\text{median reaction time}= \frac{(\ln(2))^{1/k_2}}{k_1}$$

For fluorescent data the normalization coefficients were included into the fits as follows:

$F(t)= F2-(F2-F1) \cdot e^{-(k_1 \cdot t)^{k_2}}$ for increasing signal

$F(t)= F2+(F1-F2) \cdot e^{-(k_1 \cdot t)^{k_2}}$ for decreasing signal

Where F1 and F2 are the initial and the final levels of fluorescence.
