# Supplementary material for: NusG inhibits RNA polymerase backtracking by stabilizing the minimal transcription bubble
Source: eLife. 2016 Oct 4;5:e18096. doi: 10.7554/eLife.18096 (PMC5100998; doi:10.7554/eLife.18096)
Supplement: Supplementary file 3. — DOI: http://dx.doi.org/10.7554/eLife.18096.029 [file elife-18096-supp3.pdf]

Supplementary file 3.

Analysis of TGT titration experiments in Figure 2D. All affinities are expresses as the dissociation constants. **(A)** The schematic of the experiment: the apparent affinity of TEC17 for TGT inferred from the decrease in 6-MI fluorescence upon increasing [TGT] is the product of the true affinity of the pre-translocated TEC17 for TGT and the translocation equilibrium constant. **(B)** Model employed for fitting equilibrium titration data and determining the apparent affinity of TEC17 for TGT.

A

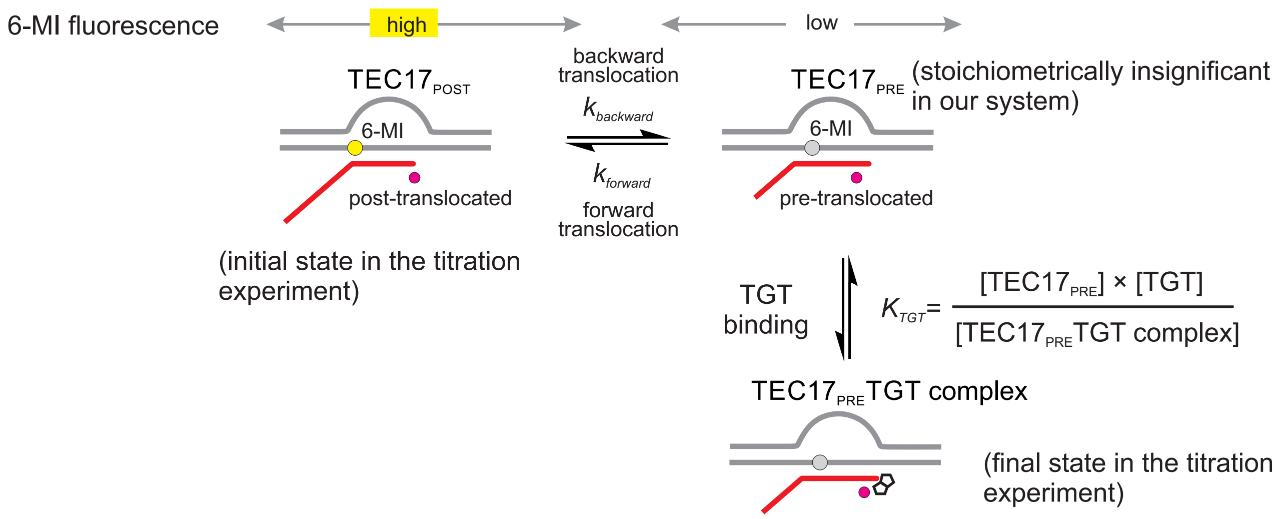

The apparent affinity of TEC17 for TGT (K_{TGT}^{app}) inferred from the decrease in 6-MI fluorescence upon increasing [TGT] is the product of the true affinity of the pre-translocated TEC17 for TGT (K_{TGT}) and the translocation equilibrium constant ($K_{translocation}$):

$$K_{TGT}^{app} = K_{TGT} \times K_{translocation} \quad \text{where } K_{translocation} = k_{forward}/k_{backward}$$

The absence of NusG effect on K_{TGT}^{app} indicates that (*i*) NusG does not affect K_{TGT} and $K_{translocation}$ or (*ii*) NusG affects both the K_{TGT} and $K_{translocation}$ in a manner that the product remains the same.

B

System of equations:

$$F = F^{TEC17\_TGT} \times TEC17\_TGT - F^{TEC17} \times (TEC17_{total} - TEC17\_TGT)$$

$$K_{TGT}^{app} = \frac{(TEC17_{total} - TEC17\_TGT) \times (TGT_{total} - TEC17\_TGT)}{TEC17\_TGT}$$

Constrains:

$0 < TEC17\_TGT < TEC17_{total}$

$0 < TEC17\_TGT < TGT_{total}$

$0 < K_{TGT}^{app}$

Independent variables: TGT_{total}, total TGT concentration

Dependent variables:

F -Fluorescence of 6-MI base in TEC17

Parameters:

K_{TGT}^{app} - the appparent equilibrium constant of TGT dissociation

F^{TEC17} -normalization coefficient (fluorescence of unligated TEC17)

F^{TEC17_TGT} -normalization coefficient (fluorescence of TGT saturated TEC17)
