# Supplementary material for: NusG inhibits RNA polymerase backtracking by stabilizing the minimal transcription bubble
Source: eLife. 2016 Oct 4;5:e18096. doi: 10.7554/eLife.18096 (PMC5100998; doi:10.7554/eLife.18096)
Supplement: Supplementary file 4. — DOI: http://dx.doi.org/10.7554/eLife.18096.030 [file elife-18096-supp4.pdf]

Supplementary file 4.
Model employed for fitting NusG binding kinetics in Figure 6C *Left*.

Initial conditions:

T=0 A = 0.2 μM B = 0.1-10 μM C = 0

TEC17 NusG TEC17_NusG complex

$$A + B \underset{k_{-1}}{\overset{k_{+1}}{\rightleftharpoons}} C$$

Rate equations (See Note 1)

$dA/dt = -A\times B\times k_{+1} + C\times k_{-1}$

$dB/dt = -A\times B\times k_{+1} + C\times k_{-1}$

$dC/dt = A\times B\times k_{+1}$

Equations for dependent variables:

SF_fluorescence=F1×A + F2×C

Independent variables: T -time

Dependent variables:
SF_fluorescence -Fluorescent traces from stopped-flow experiment

Parameters:
k_{+1} -rate of NusG binding to the TEC
k_{-1} -rate of NusG dissociation from the TEC_NusG complex

F1 -normalization coefficient for stopped-flow data
F2 -normalization coefficient for stopped-flow data

Note 1: Rate equations are uniquely defined by the reaction scheme and do not need to be explicitly specified when fitting data with Kintek Explorer.

To account for the interdependence between the binding and the dissociation rate constants we determine the lower and upper bounds of the equilibrium constant for NusG binding (expressed as the dissociation constant) as follows:

1. we fixed k_{-1} at the bestfit value and determined the upper and lower bounds for k_{+1}.
2. we calculated K_D^{NusG} using the bestfit value of k_{-1}, the upper and lower bounds for k_{+1}
3. we fixed k_{+1} at the bestfit value and determined the upper and lower bounds for k_{-1}.
4. we calculated K_D^{NusG} using the bestfit value of k_{+1}, the upper and lower bounds for k_{-1}
5. we reported the lowest lower bound and the highest upper bound from the values obtained in steps 2 and 4.
